# Examining characteristics, knowledge and regulatory practices of specialized drug shops in Sub-Saharan Africa: a systematic review of the literature

**DOI:** 10.1186/1472-6963-12-223

**Published:** 2012-07-27

**Authors:** Francis N Wafula, Eric M Miriti, Catherine A Goodman

**Affiliations:** 1 Health Systems and Social Science Research Group, KEMRI-Wellcome Trust Research Programme, Box 43640-00100, Nairobi, Kenya; 2 Department of Public Health and Policy, London School of Hygiene and Tropical Medicine, Keppel Street, London, WC1E 7HT, UK

## Abstract

**Background:**

Specialized drug shops such as pharmacies and drug shops are increasingly becoming important sources of treatment. However, knowledge on their regulatory performance is scarce. We set out to systematically review literature on the characteristics, knowledge and practices of specialized drug shops in Sub-Saharan Africa.

**Methods:**

We searched PubMed, EMBASE, WEB of Science, CAB Abstracts, PsycINFO and websites for organizations that support medicine policies and usage. We also conducted open searches using Google Scholar, and searched manually through references of retrieved articles. Our search included studies of all designs that described characteristics, knowledge and practices of specialized drug shops. Information was abstracted on authors, publication year, country and location, study design, sample size, outcomes investigated, and primary findings using a uniform checklist. Finally, we conducted a structured narrative synthesis of the main findings.

**Results:**

We obtained 61 studies, mostly from Eastern Africa, majority of which were conducted between 2006 and 2011. Outcome measures were heterogeneous and included knowledge, characteristics, and dispensing and regulatory practices. Shop location and client demand were found to strongly influence dispensing practices. Whereas shops located in urban and affluent areas were more likely to provide correct treatments, those in rural areas provided credit facilities more readily. However, the latter also charged higher prices for medicines. A vast majority of shops simply sold whatever medicines clients requested, with little history taking and counseling. Most shops also stocked popular medicines at the expense of policy recommended treatments. Treatment policies were poorly communicated overall, which partly explained why staff had poor knowledge on key aspects of treatment such as medicine dosage and side effects. Overall, very little is known on the link between regulatory enforcement and practices of specialized drug shops.

**Conclusions:**

Evidence suggests that characteristics and practices of specialized drug shops differ across rural and urban locations, and that these providers are highly responsive to client demand. However, there is a dearth in knowledge on how regulatory enforcement influences their characteristics and practices, and what strategies can be employed to strengthen the governance of the retail pharmaceutical sector.

## Background

Private medicine retailers (PMRs) are fast becoming key players in promoting access to medicines in low and middle-income countries [1-4]. Previously viewed largely as informal providers operating at the fringes of legitimacy, PMRs have gained prominence in the recent past, with recent global initiatives such as the Affordable Medicines Facility for malaria (AMFm) exploring ways of utilizing them as a vehicle for expanding coverage for essential health services. The rising interest in retailers has resulted in increased frequency of interventions targeting their performance [5,6]. In Sub-Saharan Africa (SSA), PMRs include shops that specialize in selling medicines, and those licensed to sell a limited range of pre-packaged medicines alongside groceries and other household items. The term specialized drug shops (SDSs) denotes the former category; the latter is commonly referred to in the literature as general shops [2,6]. Specialized drug shops primarily include pharmacies; legally recognized non-pharmacy drug shops (part II drug shops); unregistered drug shops; and community-owned drug shops [6]. The typology was discussed in a previous review [6].

Like other providers, SDSs are subject to regulations stipulating the minimum requirements for entry into the business and quality of care. However, widespread regulatory infringement has been reported [2,7-11]. Reviews have previously examined consumer behavior [1] and interventions for improving services provided by retailers [1,6,12]. However, no review has described regulatory practices of SDSs, despite the fact that this is the group of PMRs most often seen as an integral part of the formal health system, particularly where strategies such as the AMFm are concerned. This is the main purpose of the review.

## Methods

Original studies of all designs that described knowledge, characteristics, and practices of stand-alone SDSs in SSA were included. These included independent primary studies, evaluations conducted by non-governmental organizations, and reports commissioned by development partners. Reviews and opinion pieces were excluded, as were studies on pharmacies operating within health facilities, those looking at drug content, and those that failed to specify whether study units were SDSs or general shops. Finally, the review timeline was restricted to 20 years (1989–2009, later expanded to include studies published in 2010 and January 2011). The time cap was added to ensure the capture of studies reporting relatively recent SDS experiences.

A search strategy that combines population and geographical location search terms was then developed and applied to PubMed, EMBASE, CAB Abstracts and PsycINFO (Table 1). We searched websites for the International Network for the Rational Use of Drugs (INRUD), e-Drug, WHO Essential Medicines and Management Sciences for Health, for project reports and references to articles that may have some relevance. Additionally, we conducted cited-reference searches in the Web of Science database to identify studies citing other relevant studies, and did open Google Scholar searches using the terms outlined in Table 1. Finally, references of retrieved articles were searched manually for other relevant studies, and all references imported into an EndNote X library. Duplicated articles were removed from the library.

**Table 1:** Search terms used in searching electronic databases

***Group A: Target population [combined by ‘OR’]***		***Group B: Geographical location [combined by ‘OR’]***
drug retailer* OR medicine retailer* OR pharmacy OR pharmacies OR drug shop* OR medicine shop* OR drug seller* OR medicine seller* OR drug vendor* OR medicine vendor* OR drug store* OR medicine store* OR pharmacies [MeSH] OR drug dispensing shop* OR medicine dispensing shop* OR medicine outlet* OR drug outlet* OR medicine dealer*	*AND*	*Angola OR Benin OR Burkina Faso OR Burundi OR Cape Verde OR Central African Republic OR Chad OR Comoros OR Democratic Republic of Congo OR Djibouti OR Equatorial Guinea OR Eritrea OR Ethiopia OR Gambia OR Guinea OR Guinea-Bissau OR Lesotho OR Liberia OR Madagascar OR Malawi OR Mali OR Mauritania OR Mozambique OR Niger OR Rwanda OR Sao Tome and Principe OR Senegal OR Sierra Leone OR Somalia OR Sudan OR Tanzania OR Togo OR Uganda OR Zambia OR Cameroon OR Congo OR Côte d'Ivoire OR Ivory Coast OR Ghana OR Kenya OR Nigeria OR Zimbabwe OR Namibia OR Swaziland OR Botswana OR Gabon OR Mauritius OR South Africa OR Seychelles OR Africa South of the Sahara [MeSH] OR Sub-Saharan Africa*

**Figure 1:**
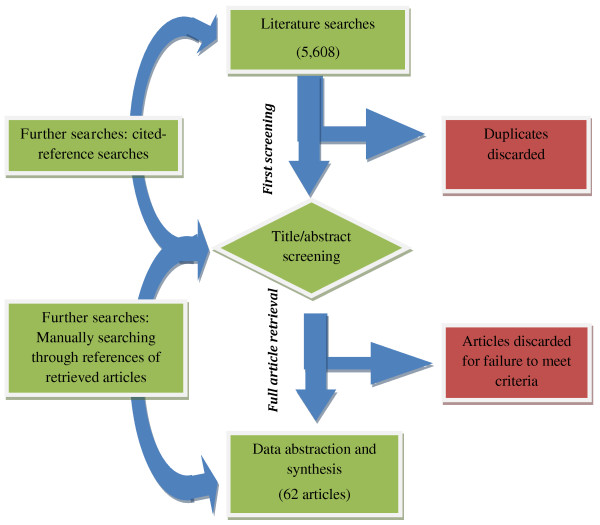
Summary of the literature searching and reviewing processes.

A total of 5,608 articles were identified (Figure 1) (Last search: 11^th^ February 2011), and screened separately by 2 reviewers (FW and EM) using the inclusion criteria outlined above. Articles that did not meet the requirements were removed from the list. Where there was lack of clarity, the 2 reviewers (FW and EM) discussed the article’s content, and made a joint decision on whether or not it should be included. At the end of the process, 62 articles were retrieved and included in the review. Information was abstracted on author, year of publication, country and location (rural or urban), study design and sample size, outcomes investigated, and the key findings (Table 2). For intervention studies, baseline values - rather than post-intervention data - were abstracted, with the underlying thought that pre-intervention values were a better reflection of typical SDS practices within the respective regions. Similarly, data was abstracted from the control group in post-hoc evaluations. The studies were heterogeneous in terms of outcomes reported, thus rendering a meta-analysis of results inappropriate. Consequently, a structured narrative synthesis was undertaken.

**Table 2:** Summary of study characteristics and findings

**Ref No**	**Author (year), country of study**	**Number and type of shops (staff)**	**Study design (SCM)**	**Areas investigated**	**Major findings**
*66*	*Abula et al (2006) Ethiopia, urban*	*20 Pharmacies*	*Cross-sectional survey*	*Drug sources, sources of information, handling of clients requesting for partial doses, and patient referral*	*-Poor access to drug information; 80% get no up-to-date information on medicines (and rely on inserts instead)*
					*-All staff reported referring patients when required*
					*-40% accept partial dispensing*
*70*	*ACTwatch (2009) Benin, urban and rural*	*35 pharmacies (30 registered and 5 rural outpost pharmacies)*	*Cross-sectional survey*	*Availability, price, and volume of antimalarials sold*	*-77% stocked the first-line ACT, with 87% stocking non-artemisinin antimalarials. Diagnostic tests*
				*Knowledge of providers*	*- Pharmacy charged higher prices compared to other providers, but sold lower volumes overall*
*30*	*ACTwatch (2009) D.R Congo, urban and rural*	*31 pharmacies and 470 drug stores*	*Cross-sectional survey*	*Availability, price, and volume of antimalarials sold*	*-Both shops rarely stocked first-line (only 20-25%), but most had artemisinin monotherapy in stock*
					*-Both shop types did not generally do diagnostic tests*
				*Knowledge and perceptions of providers*	*-Pharmacies charged lowest prices for ACTs*
					*-Drug stores sold the most, but had least knowledge*
*33*	*ACTwatch (2009) Madagascar, urban and rural*	*80 registered pharmaciesand 162 rural pharmacies*	*Cross-sectional survey*	*Availability, price, and volume of antimalarials sold*	*-Only half stocked the first-line; majority had non-artemisinin drugs, and did not do diagnostic tests*
					*-Pharmacies charged highest prices for ACTs by far*
				*Knowledge and perceptions of providers*	*-They sold the highest volume of medicines, but only 59% knew the recommended first-line treatment*
*29*	*ACTwatch (2009) Nigeria, urban and rural*	*38 pharmacies and 305 patent medicine stores*	*Cross-sectional survey*	*Availability, price, and volume of antimalarials sold*	*-74% of pharmacies had first-line, compared to 8% for PMVs*
					*-Both shop types did not generally do diagnostic tests*
				*Knowledge and perceptions of providers*	*-Pharmacies charged highest prices for recommended ACTs*
					*-PMVs sold the most, but had least knowledge on treatment*
*32*	*ACTwatch (2009) Uganda, urban and rural*	*65 pharmacies and 188 drug stores*	*Cross-sectional survey*	*Availability, price, and volume of antimalarials sold*	*-57% of pharmacies had first-line; only 4% of drug shops*
				*Knowledge and perceptions of providers*	*-Both shop types rarely carried out diagnostic tests*
					*-Pharmacies charged highest prices for recommended ACTs*
					*-Drug stores sold the highest volume of medicines, but only 60% knew the recommended first-line treatment*
*31*	*ACTwatch (2009) Zambia, urban and rural*	*49 pharmacies and 130 drug stores*	*Cross-sectional survey*	*Availability, price, and volume of antimalarials sold*	*-Most pharmacies sold ACTs, but only 6% of drug shops did –Non-registered ACT also very common in pharmacies*
					*-32% of pharmacies did diagnostic tests, but not drug stores*
				*Knowledge and perceptions of providers*	*-Pharmacy ACT prices were higher than drug stores*
					*-Drug stores had much lower knowledge on first-line drug*
*25*	*Adikwu, M. U (1996) Nigeria, peri-urban*	*46 Patent medicine stores*	*Cross-sectional survey*	*Regulatory inspections, characteristics and knowledge of dispensers*	*-All staff aware of the law governing retailers in Nigeria*
					*-Main suppliers were pharmaceutical representatives*
					*-Sales boys and girls used in patent medicine stores*
*68*	*Adisa et al (2006) Nigeria, urban*	*50 Pharmacies*	*Cross-sectional survey*	*Knowledge on characteristics, ethics and perceived responsibility with regard to phyto-pharmaceuticals*	*-3/4 scored below 53% on knowledge about the drugs*
					*-1/3 had training on phyto-pharmaceuticals*
					*-3/4 felt such drugs need Regulatory Authority governance*
					*-3/4 felt pharmacists qualified to handle such medicines*
*56*	*Adu-Sarkodie et al (2000) Ghana, urban*	*48 pharmacies*	*Baseline survey before intervention (SCM)*	*-Management practices for STI clients*	*-About half of the shops took patient history before selling*
					*-Only one-fifth counseled clients on partner notification*
					*-Only one-fifth offered the recommended medicines*
					*-Only 13% advised STI clients to use condoms*
*34*	*Amin et al, 2005, Kenya, rural*	*20 Pharmacies*	*Cross-sectional survey*	*-Drug sales practices*	*-Unregistered drugs found in some pharmacies*
					*-Majority of drugs not within the registration period*
				*-Regulation and registration policy framework*	*-New first-line AL found in only 11% of pharmacies*
					*-Sourced drugs mainly from wholesalers, never from vendors*
				*-Adherence, stocking practices*	
*60*	*Berih et al (1989) Sudan, urban*	*63 pharmacies*	*Cross-sectional survey (SCM)*	*Drug dispensing practices*	*-Only 5% recommended ORS despite wide availability, with 2/3 recommending antimicrobials which cost 4 times more*
					*-63% took history before treatment; these were more likely to refer child and less likely to recommend antimicrobials*
					*-ORS use not related to availability or history taking*
*47*	*Blanchard et al (2005) South Africa, urban*	*28 Pharmacies*	*Cross-sectional survey*	*Knowledge and attitudes of pharmacists towards providing emergency contraception*	*-Nearly all pharmacists sold at least one of the two ECPs*
					*-Most were familiar with contraceptive indication and side effects, but felt they should not be given to under 18s*
					*-About 80% were willing to display promotional materials*
*62*	*Brieger et al (2004) Nigeria, rural and urban*	*149 patent medicine shops; (820 observations)*	*Cross sectional survey*	*Nature of interaction between patent medicine vendors and clerks and clients*	*-Quarter of clients shared problems with staff*
					*-69% sold requested medicines, 30% gave treatment suggestions, 21% gave instructions on medication use, and 19% asked questions about the illness*
*49*	*Brieger et al (2007) Nigeria, rural*	*12 patent medicine sellers*	*Qualitative study*	*-Medicine sellers perceptions of consumer color preferences for medicines*	*-Sellers linked color to effects; yellow associated with malaria because of symptoms of yellow urine and eyes*
					*-Sellers had low opinion of white colored medicines*
*73*	*Buabeng et al (2008) Ghana, urban and rural*	*35 pharmacies and 64 licensed chemical shops*	*Cross-sectional survey*	*-Availability of antimalarials*	*-SPs the most available antimalarial; ACTs less available, especially in chemical shops and rural areas*
				*-Policy adherence when choosing drugs to dispense*	*-Poor adherence to policy guidelines when choosing drugs*
					*-Unregistered and unrecommended drugs stocked*
				*-Types of medicines stocked*	
*51*	*Buabeng et al (2010a) Ghana, urban and rural*	*35 pharmacies and 64 licensed chemical shops*	*Cross-sectional survey*	*-Characteristics of staff*	*-45% of pharmacies had professional staff (pharmaceutical or nursing) compared to 5% for LCSs; 24% of pharmacy staff could treat malaria with ACTs compared to 6% LCSs*
				*-Knowledge of staff on malaria*	*-76% of pharmacy staff knew symptoms of complicated malaria compared to 43% in LCS*
				*-Practices on malaria prevention*	
*65*	*Buabeng, K. O (2010b) Ghana, urban and rural*	*35 pharmacies and 64 licensed chemical shops*	*Cross-sectional survey*	*-Suitability of premises for malaria services provision*	*-Most pharmacies clean and well lit compared to LCSs*
					*-74% pharmacies had counseling area versus 19% of LCS*
					*-88% pharmacies had fridge versus 22% of LCSs*
					*-All pharmacies kept some records versus 47% of LCSs*
					*-More pharmacies than LCSs had reference materials*
*40*	*Cohen et al (2010) Tanzania, rural*	*226 part II drug shops*	*Cross-sectional survey*	*Range and patterns of availability of antimalarials vis-à-vis geographical and socio-economic determinants*	*-ACTs stocked more in shops located nearer towns and/or nearer other shops and in more populous areas (p<0.01)*
					*-Shops near ACT-stocking facilities more likely to stock the non-recommended SP medicines (p<0.01). Remote shops more likely to sell antipyretics for fever than antimalarial*
*20*	*Fayemi et al (2010) Nigeria, rural*	*97 Patent medicine vendors*	*Cross-sectional survey*	*-knowledge, dispensing practices, and referral for emergency contraceptives*	*-One-third not aware of ECPs; only half knew that ECPs could prevent pregnancy*
					*-Only half had referral systems for ECP clients*
*58*	*Goel et al (1996) Kenya, rural and urban*	*91 Pharmacies*	*Cross-sectional survey (SCM used)*	*Influence of geographical location and knowledge on prescribing practices for diarrhea*	*-No clear relationship between knowledge and prescribing*
					*-Correct treatment odds higher in high SES urban areas -*
					*-Women more likely to receive appropriate treatment for diarrhea in their children*
*28*	*Goodman (2004) Tanzania, rural*	*43 Part II drug shops*	*Cross-sectional survey*	*Range and sources of antipyretics, antibiotics and antimalarials*	*-Nearly all drug shops had fever and malaria medicines, and nearly two-thirds had antibiotics*
					*-87% got drugs from drug wholesaler or part I pharmacy*
*54*	*Goodman et al (2007) Tanzania, rural*	*30 part II drug stores*	*Cross-sectional survey*	*Compliance with regulations*	*-Majority displayed permits, but some permits belonged to staff not attached to the shop. Majority also stocked and sold prescription medicines against regulatory requirements*
				*Likely causes of regulatory infringement*	*-Poor compliance cause by low knowledge, inadequate inspections, and tacit permission from regulatory enforcers*
*22*	*Greer et al (2004) Nigeria, rural*	*245 patent medicine vendors*	*Baseline survey before intervention (SCM)*	*-Characteristics and knowledge of PMV on malaria and other diseases*	*-57, 28% had secondary or tertiary education respectively*
					*-Stability in employment; 80% had worked there for over 1 year, 54% more than 4 years, 24% for more than 10 years*
					*-21% knew ITNs, but only 5% recommended to clients*
*22*	*Greer et al (2004) Uganda, rural*		*Baseline survey before intervention (SCM)*	*-Management of malaria, ARI and diarrhea*	*-Majority took basic patient history appropriately*
					*-Nearly all recommended wrong medicine and dose*
					*-Only 8% explained how drug should be taken*
*42*	*Hera (2006) Tanzania, rural and peri-urban*	*25 ADDOs*	*Post-hoc ADDOs programme evaluation*	*Availability, affordability and quality of drugs, and the quality of dispensing services*	*Better availability but higher prices of drugs compared to public facilities. Rural shops sold at higher prices, but disparities among ADDOs in prices. Amoxicillin, quinine and ORS dispensed inappropriately*
*17*	*Hetzel et al (2006) Tanzania, rural*	*10 drug stores before, 19 after*	*Cross-sectional surveys*	*-Availability and access to drugs before and after policy change from chloroquine as first line to SP*	*-Number of shops stocking drugs almost doubled.*
					*-First-line drug (SP) had better availability in 2004 than did CQ (previous first-line) in 2001 (in drug shops)*
*23*	*Hetzel et al (2008) Tanzania, rural*	*29 part II drug stores*	*Cross sectional survey (SCM used)*	*-Knowledge on malaria treatment*	*-Drug shops had better knowledge than general shops*
				*-drug prescribing practices*	*-Mystery shoppers likely to get appropriate treatment in drug shops but at higher price.*
					*-Adults more likely to have an anti-malarial sold to them*
*52*	*Igun (1994) Nigeria, rural*	*58 pharmacies and 77 patent medicine stores*	*-Cross-sectional survey (SCM used)*	*-Knowledge and prescribing practices for watery and bloody diarrhea*	*-70% of staff said they would give ORT for diarrhea, but only 7% actually gave, the rest giving drugs*
					*-57% stated they give ORT only, but 90% of these providers gave drugs only for the diarrhea*
*13*	*Jacobs et al (2004) Uganda, rural and peri-urban*	*141 drug shops*	*Cross-sectional survey*	*-Management of urethral discharge in men, treatment outcomes, and patients’ perception of quality of care*	*-14% of patients treated according to national guidelines, but 11% managed properly*
					*-55% told to use condom or refer partner*
					*- 38% cure rate*
*41*	*Karim et al (1996) South Africa, urban*	*10 pharmacies*	*Records review*	*-Generic substitution*	*-45.7% of all prescription had generic equivalents*
				*-Cost analysis of prescriptions for branded and generic drugs*	*- The branded price was 10% higher than the generic price*
					*-Pharmacists only substituted 14% of drugs for generics*
					*-7% of costs can be saved through generic substitution*
*74*	*Kachur et al (2006a) Tanzania, urban*	*29 part I pharmacies*	*Cross-sectional survey*	*-Availability, packaging and labeling of artemisinin-containing products*	*- 89% of artemisinin drugs identified were monotherapies*
					*-All products sold as prepackaged unit doses*
					*-All drugs obtained in manufacturers' original packaging*
*69*	*Kachur et al, (2006b) Tanzania, rural*	*10 specialist drug stores*	*Cross-sectional survey*	*-Prevalence of malaria parasitemia among clients*	*-17% of febrile visits resulted in buying of an antimalarial*
				*-Characteristics of malari*	*-An antipyretic as obtained in 77% visits, with most clients not getting malaria-specific treatment when warranted*
				*a and fever medicines buyers*	*-Education linked to better buying of antimalarials*
*43*	*Kagashe et al (2011) Tanzania, urban*	*70 pharmacies*	*Cross-sectional survey*	*-Dispensing practices for antibiotics, and other drugs*	*-45% dispensed on client request, 32% on dispenser recommendation, and only 23% on prescription*
					*-Antibiotics given inappropriately and in partial doses*
*21*	*Kwena et al (2008) Kenya, urban*	*50 pharmacies*	*Cross-sectional survey (SCM)*	*-Characteristics of providers managing STI patients*	*-Only 10% of clients were offered appropriate treatment according to the government’s STI management guidelines*
				*-Knowledge on management of STIs, compliance to guidelines*	*-74% of pharmacy staff reported that some customers cannot raise all money for medicines prescribed*
*63*	*Liambila et al (2010) Kenya, urban*	*20 pharmacies*	*Survey before intervention (SCM)*	*-Counseling and dispensing practices for clients seeking emergency contraception*	*-About half of staff gave additional information on EC*
					*-Only 12.5 gave regular family planning advice*
					*-Only 5% talked about STIs/HIV*
*26*	*Maiga et al (2003) Mali, urban*	*11 Pharmacies*	*Records review*	*Drug prescription and selling practices*	*-Most purchases made without prescription were generics*
					*-Transactions were more costly in pharmacies than public health facilities (prices higher by 68%)*
*72*	*Maiga et al (2010) Mali, urban*	*30 Private drug stores*	*Survey before intervention*	*Availability and prices for essential medicines*	*-* A*vailability of 49 essential drugs was 66.1% among the retail pharmacies*
					*-Retail prices were 25% higher than recommended*
*35*	*Manirakiza et al (2010) Central African Republic, urban*	*15 Private drug stores and 60 non-official drug shops*	*Cross-sectional survey*	*-Availability of antimalarials*	*-87% of drug stores sold artemisinin monotherapy*
				*-Performance of staff in management of malaria*	*-70 and 93% of drug stores and unofficial drug shops sold the unrecommended chloroquine*
					*-Chloroquine was not supplied by the official wholesalers*
					*-SP, the official treatment, was available in all drug stores, but unavailable in 84% of unofficial drug shops*
*44*	*Massele et al (1993) Tanzania, urban*	*20 drug shops*	*Cross-sectional survey*	*-Knowledge and treatment practices for malaria*	*-Knowledge of drug sellers on sign and symptoms was adequate, but 45% did not know the correct drug dose*
					*-Only 30% of patients knew the correct dose*
*61*	*Mayhew et al (2001) Ghana, urban*	*248 pharmacies*	*Survey before intervention (SCM)*	*-Characteristics of shops, staff and clients*	*-Only 34% of staff had university qualification*
				*-STI management of clients*	*-60% of STI clients visit without a prescription*
					*-Only 17% used national guidelines to treat STIs*
*27*	*Mazzilli et al, 2009 Somaliland, urban and rural*	*83 pharmacies*	*Cross-sectional survey*	*-Characteristics of shops, staff*	*-No pharmacists found. Nurses own most pharmacies*
					*-Pharmacies offered injections and diagnostic tests*
				*-Knowledge about drugs*	*-Majority knew drugs through self study and experience*
				*-Services and drugs offered*	*-Majority sold medicines without prescription*
*7*	*Minzi et al (2008) Tanzania, urban*	*551 pharmacies*	*Cross-sectional survey*	*-Types of antimalarials stocked at the pharmacies*	*-None of had been involved in preparation of guidelines*
					*-Poor knowledge on pediatric dosages*
				*-Awareness on new malaria treatment guidelines*	*-49% of pharmacies still stocked chloroquine*
					*-Only 30/7% knew dose regimen of SP/AL respectively*
*18*	*Murray et al (1998) Eritrea (rural)*	*59 rural drug vendors*	*Cross-sectional survey (SCM)*	*-Knowledge and treatment practices for diarrhea and respiratory infection*	*-41% gave correct treatment for respiratory infection*
					*-Only 38% of clients knew correct dosing for antibiotics*
					*-63% knew how to treat diarrhea, but only 10% did so*
*14*	*Nakyanzi et al (2010) Uganda, urban*	*32 pharmacies*	*Cross-sectional survey*	*-Range and characteristics of medicines that expire within pharmacies*	*-75% had gotten low-price/donations of near-expiry drugs;* 56% *had disposed of expired drugs; 44% had returned drugs to suppliers, and 28% had customers return drugs*
					*-Policy change in treatment linked to expiry of medicines*
*36*	*Noor et al (2009) Somalia, rural*	*194 pharmacies*	*Cross-sectional survey*	*-Availability of malaria drugs and diagnostic services*	*-Over 30% do diagnosis; CQ found in 53% of shops, with 9% providing SP. Artemisinin monotherapy was found in 14% of shops, with one area having 42% availability*
				*-Prescribing for malaria*	*-ACT only found in 9% of shops (2 years after new policy)*
*45*	*Nshakira et al (2002) Uganda, rural*	*2 drug shops*	*Cross –sectional survey*	*-Drugs and instructions given to children under five*	*- 75% of medication was bought in shops in community*
				*-Instructions given to caretakers about dosage*	*-All study sites had a range of ant malarial drugs in stock -An average of 3.2 drugs was dispensed per child*
*59*	*Nsimba (2007) Tanzania, rural*	*4 pharmacies and 39 drug stores*	*Cross-sectional survey (SCM used)*	*-Dispensing practices for ORS, antimalarials and antibiotics*	*-Antibiotics overused in both urban and rural settings*
					*-Use of branded drugs more common than generics*
				*-Training on selectively prescribing*	*-ORS rarely prescribed. Antibiotics inappropriately dispensed for watery diarrhea in almost half of the cases*
*8*	*Ntambwe et al (1994) Zaire (now D.R. Congo), rural*	*44 pharmacies*	*Cross-sectional survey*	*-Characteristics of premises, staff, and availability of permit*	*-None run by pharmacist or assistant. 30% run by nurses; 71% run by untrained persons; all shops owned by traders*
				*-Compliance with dispensing and recording regulations*	*-None had a permit, and none kept prescription registers*
					*-87% sold drugs without prescription*
*64*	*Nyazema et al (2007) Zimbabwe, urban*	*63 pharmacies*	*Cross –sectional survey (SCM used)*	*Sale of antibiotics without prescription, and provision of treatment advice according to acceptable standards*	*-69% said they can’t sell antibiotics without prescription*
					*-Actual sale of antibiotics without prescription was low*
					*-Few respondents performed well regarding provision of information and advice in relation to treatment guidelines*
*9*	*Ojuawo et al (1993) Nigeria, urban*	*75 Patent medicine sellers*	*Cross –sectional survey (SCM used)*	*-Knowledge, treatment and referral practices for diarrhoea*	*-33% were owners. 30% of employees were primary school children. Majority of staff did not ask questions about diarrhea, and lacked knowledge on ORT*
					*-Diarrhea drugs were recommended by all respondents*
*48*	*Okeke et al (2006) Nigeria, rural*	*13 patent medicine sellers*	*Survey before intervention*	*Knowledge, beliefs, treatment practices, and referral for mild and severe malaria*	*-Majority of sellers not health professionals*
					*-Poor knowledge, dispensing, and referral for malaria*
					*-Advice and information rarely given to care-givers*
					*-62% dispensed what clients demand; only 15% took history*
*37*	*Onwujekwe et al (2010) Nigeria, rural*	*11 pharmacies and 137 patent medicine sellers*	*Cross-sectional survey*	*-Characteristics of providers*	*-14% of PMVs sold medicines without any diagnostic steps compared to 5% for pharmacies. A high proportion of both shops used history to confirm diagnosis for malaria*
				*-Knowledge and management of malaria*	*-More pharmacies sold artemisinin monotherapies*
*53*	*Oreagba et al (2010) Nigeria, urban*	*400 pharmacies*	*Cross-sectional survey*	*-Knowledge, perceptions and practice around pharmacovigilance*	*-55% had heard of pharmacovigilance; 18% could define it*
					*-Only 3% reported adverse drug reactions to the authority*
					*-45% did not report because they did not know how*
*46*	*Oshiname et al (1992) Nigeria, rural*	*37 patent medicine vendors*	*Baseline before intervention*	*-Knowledge on symptoms, counseling and management of malaria and other diseases*	*- 70% knew the correct malaria drug, but only one knew the correct dosage for a 3 year old child*
					*-39% would sell medicine to a child*
*19*	*RPM Plus (2006), Tanzania urban and rural*	*58 ADDOs*	*Cross –sectional survey (SCM)*	*Knowledge and management of major childhood illnesses, and availability of key drugs*	*-Poor knowledge on treatment choices for diarrhea and ARI*
					*-Low adherence to national guidelines for treating ARI, malaria and diarrhea*
*16*	*Russo et al (2010) Mozambique urban*	*34 private pharmacies*	*Cross-sectional survey*	*-Prices for generic and branded medicines*	*-Huge price variations across pharmacies despite price regulation*
					*-Pharmacies adjust prices depending on the market demand*
*38*	*Sabot et al (2009) Tanzania, rural*		*Baseline survey before intervention*	*-Availability and dispensing practices for ACTs*	*-Nearly none of the shops stocked the recommended ACTs before the intervention (subsidy) was introduced*
					*-As a result, most shops did not suggest/offer ACTs to clients, choosing to instead offer SPs*
*11*	*Tumwikirize et al (2004) Uganda, urban*	*28 pharmacies and 169 drug stores*	*Cross-sectional survey*	*-Characteristics, knowledge and dispensing practices for ARIs*	*-Most staff had training background in nursing*
					*-Low knowledge on ARI symptoms and management*
					*-Inappropriate dispensing of antibiotics very common*
*39*	*van den Boogaard et al (2010) Tanzania, rural*	*14 pharmacies and 15 drug shops*	*Cross-sectional survey*	*-Availability and sale of floroquinolones and other antibiotics*	*-All drug shops illegally stocked and sold antibiotics*
					*-Floroquinolones widely available in shops, raising concerns over resistance*
*24*	*Viberg et al (2009) Tanzania, rural*	*94 pharmacies, drug stores, and ADDOs*	*Cross –sectional survey (SCM used)*	*-STI management practices*	*-74% of drug sellers stated they had no STI-related drugs in the stock, but 78% and 63% gave male and female simulated clients the medicines, mostly antibiotics*
				*-Knowledge on dose, side effects*	
				*-Drug dispensing practices*	*-Most drugs dispensed were the recommended, though incomplete management, incorrect dosages, lack of advice, and poor history-taking were common*
				*-Knowledge on regulation*	
*50*	*Viberg et al (2010) Tanzania, rural*	*75 drug sellers*	*Cross-sectional survey*	*-Knowledge and perceptions on antibiotic use and resistance*	*-79% of drug sellers knew antibiotics treat bacterial infections; 24% of these sellers also thought antibiotics could treat viral infections*
					*-72% had heard about antibiotic resistance*

## Results

**Figure 2:**
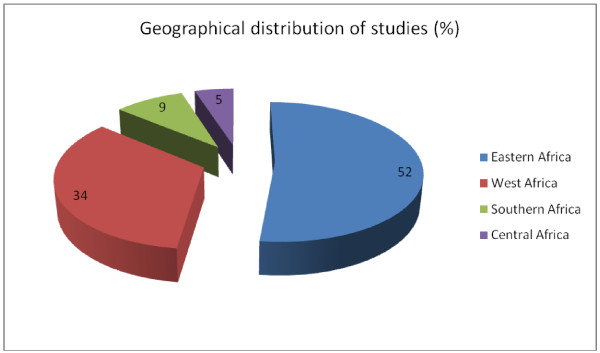
Distribution of studies by geographical location.

**Figure 3:**
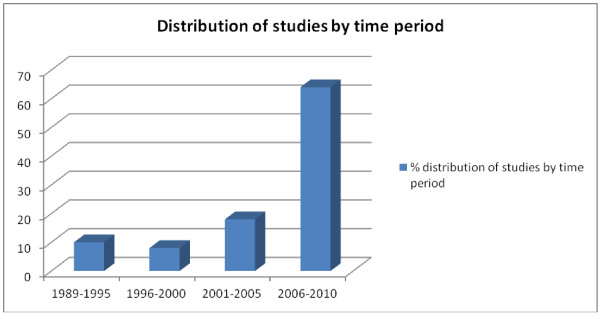
Distribution of studies across different time periods.

**Figure 4:**
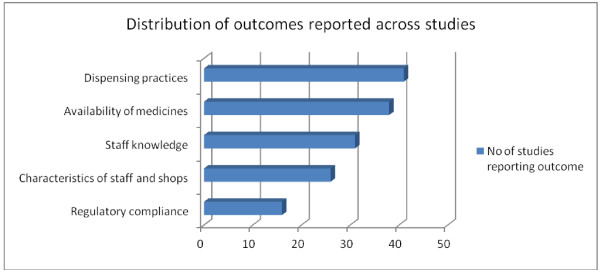
Distribution of the main outcomes reported.

A vast majority of the studies were descriptive cross-sectional surveys. Studies from the Eastern African region contributed more than half of all studies reviewed (Figure 2), with Tanzania alone having 17 studies. Nigeria had the highest number in West Africa, with 13 studies. The Southern and Central African regions had few studies overall. More studies were conducted in rural/peri-urban areas compared to urban locations (42 compared to 34), with a few studies including both locations. A pattern showing an increase in the number of studies over recent years emerged, with nearly two-thirds of the studies reviewed being conducted over the last 5 year band (2006–2011) (Figure 3). Studies reported a wide range of outcomes including dispensing practices (41 studies), availability of medicines [13], staff knowledge [14], staff and/or shop characteristics [15], and adherence to policy guidelines or regulatory requirements (16 studies) (Figure 4).

### Characteristics of staff, commodities stocked, and shops

A wide variety of SDSs were encountered, including pharmacies and non-pharmacy outlets. Non-pharmacy SDSs included accredited drug dispensing outlets (ADDOs) and the older part II drug stores (also referred to as ‘duka la dawa baridi’ in Swahili) in Tanzania, licensed chemical sellers (LCSs) in Ghana, patent medicine vendors (PMVs) in Nigeria, rural drug vendors in Eritrea, and drug shops in Uganda among others. Pharmacies were found mainly in urban areas, with the non-pharmacy SDSs operating more in rural areas [16-19]. Specialized drug shops were more likely to be found in affluent and highly populated areas overall. In Tanzania and Eritrea, for instance, a positive correlation was reported between the number of medicine outlets and village or town size [20,21]. As distribution of health facilities follows similar patterns, there is danger of low density areas having neither a health facility nor an SDS; indeed, half of ADDOs in Tanzania were located less than one kilometer from a health facility [22].

Shop ownership varied across studies, but rural SDSs were more likely to have owners or relatives working as staff, whereas urban shops had a higher likelihood of having employees. This phenomenon, seen in Kenya and Nigeria [9,23,24], may explain Greer *et al’*s observation of relatively low staff turnover among rural PMVs in Nigeria, where over half of staff had worked at the same shop for over 4 years [25]. Although staff qualification varied substantially, those working in pharmacies and part II drug shops generally held higher qualifications compared to those working in PMVs. In Kenyan pharmacies for instance, nearly all staff were found to have tertiary health-related training [24]; similarly, nearly all staff working in Tanzanian part II drug stores and Ugandan drug shops were reported to have some health-related training [11,26,27]. The majority of staff were nurse aides. In Nigeria on the other hand, PMVs were found employing children under the legally mandated age for dispensing (21 years), most being school going children [9,28]. However, pharmacies in some areas were also guilty of hiring staff with no qualifications after obtaining registration; in Mali, for instance, majority of individuals dispensing in pharmacies were found to have no professional qualifications [15]. Although pharmacies usually require pharmacy-trained staff, Somaliland was a notable exception; there, medical doctors and nurses were allowed to run pharmacies due to an acute shortage of pharmaceutical staff [29].

SDSs were well stocked overall, although urban pharmacies were generally better stocked compared to their rural counterparts. In Somaliland for instance, urban and rural pharmacies had 70% and 40% of specified essential medicines respectively [29]. Drug shops were found to have good availability of a narrower range of medicines, usually fever and malaria drugs [20,30]. However, good availability did not always mean good geographical access to recommended treatments. This was particularly seen in the case of antimalarials, where the more widely available drugs were not the recommended first-line treatments. This trend was seen in Kenya, Uganda, Tanzania, Madagascar, Nigeria, Zambia, Somalia and the Central African Republic [13,14,31-39]. In Tanzania, pharmacies were more likely to stock non-recommended antimalarials if they were located near health facilities that stocked the recommended drugs [40]. Drug expiry was a problem in some areas but not others. In Uganda, nearly half of the shops reported cases of drugs expiring, whereas only one case was observed in Somaliland [17,29]. Generic substitution was rarely done; in South Africa for instance, only 14% of pharmacists substituted branded drugs, despite the fact that nearly half of prescribed medicines had generic equivalents [41].

### Knowledge and perceptions

Staff knowledge was assessed at two broad levels; knowledge specific to treatment, and knowledge on wider aspects of practice such as treatment policies. Knowledge on drug specific aspects was found to vary substantially, although certain patterns were consistent across studies. Majority of staff knew what drugs to give for tracer conditions, but had less knowledge on appropriate dosing. This was seen in Tanzania [7,26,27,42-44], Uganda [25,45], Eritrea [21], and Nigeria [31,46]. Misconceptions were also reported: for instance, most South African pharmacists erroneously believed that emergency contraceptive pills (ECPs) posed health risks if used repeatedly [47], majority of Nigerian PMVs believed fever medicines could cure malaria on their own [48], and that white colored medicines were less efficacious overall [49], and nearly one-fifth of Tanzanian drug sellers thought that antibiotics could cure viral infections [50]. Where pharmacies and non-pharmacy SDSs were compared, the former performed better overall. In Ghana for instance, three quarters of pharmacy staff knew symptoms of complicated malaria compared to 43% for LCSs [51]. A few studies went further and compared knowledge to practice. In Nigeria, 70% of pharmacies and PMVs knew oral rehydration therapy (ORT) to be the treatment of choice for diarrhea and yet a vast majority sold antidiarrhoeals and antibiotics instead [52], whereas only 10% of Eritrean rural drug vendors gave correct diarrhea treatment, despite the fact that 63% knew the correct treatment [21]. Similarly, amodiaquine and quinine were sold as much as sulphur medicines in Tanzanian drug stores, despite staff knowing that only the latter was recommended for uncomplicated malaria at the time [26]. Discordances between knowledge and practice extended beyond treatment; in another Tanzanian study for instance, only a few ADDOs advised malaria clients to sleep under an ITN, despite the majority knowing about the effectiveness of bednets as a preventive strategy [22]. Similarly, although 55% of Nigerian pharmacies knew about pharmacovigilance, only one fifth of pharmacies had actually made an attempt to report an ADR to relevant authorities [53]. Knowledge on broader policy issues was good overall, with all PMVs in a Nigerian survey knowing about prescription-only medicine status [28], and majority of Tanzanian drug stores being aware that they were only allowed to sell certain medicine categories, although there was considerable confusion on which malaria medicines fell in this category [54]. There were rural-urban differences in knowledge on policy issues in Somaliland, with rural pharmacies being less aware of the difference between prescription and non-prescription drugs, and rationale behind restricting the former [29].

### Dispensing practices

Dispensing practices were grouped into three categories; treatment practices, communication, and client referral. Most studies touched on more than one category, with some exploring associations between dispensing practices and factors such as shop location, staff qualification, and client characteristics (age, sex, level of education and familiarity to the attendant).

Studies assessing treatment practices looked at a wide range of factors, including drug selection, adherence to treatment protocols and dispensing without prescription. The degree to which SDSs adhered to treatment guidelines was varied but low overall. Adherence to national guidelines when managing STI clients was low in Kenya (27% adherence) [55], Ghana (20-23%) [56,57], and Uganda (8-14%) [16,25]. Inappropriate dispensing was also observed for diarrhoea in Kenya [58], Tanzania [43,59], Nigeria [9,52] and Sudan [60]. However, good performers were also noted; in Tanzania for instance, 80 - 90% of drug stores dispense drugs recommended in national guidelines for STIs, although the authors noted the irony in the fact that the shops were not allowed to stock the medicines in the first place [27]. It appeared that some respondents were not completely honest in their responses, possibly because they knew that they were engaged in unlawful behavior. In another Tanzanian study for instance, three-quarters of drug stores reported not having medicines for sexually transmitted infections (STIs) in stock, and yet these drugs were dispensed to 63% and 78% of female and male mystery shoppers respectively [27].

Like treatment practices, communication with clients gave mixed results. Shops were more likely to communicate basic medication instructions after selling medicines than take patient history before treatment, or offer advice on alternative management strategies. Basic advice and treatment instructions (how and when to take medicines) were given in Ghanaian pharmacies (89% of clients), and Tanzanian drug stores and ADDOs (89 and 93% respectively) [22,27,61], but rarely done in Kenyan pharmacies (17%) and Nigerian PMVs (10-13%) [9,55]. More technical aspects such as side effects and contra-indications were rarely discussed among drug stores in Tanzania and PMVs in Nigeria [22,27,48]. Clients who requested specific drugs, or those who failed to disclose the purpose of purchasing medicines were less likely to receive advice in Nigeria and Uganda [16,62]. Pharmacies on the other hand performed relatively well in counseling, with nearly two-thirds of South African pharmacies discussing prevention of pregnancy with contraception clients [47], and majority of Ghanaian and Kenyan pharmacies counseling STI clients on the importance of partner treatment (81 and 84% respectively) [55,61]. Pharmacies also offered more privacy during counseling, as seen in Zimbabwe, Kenya and Somaliland [29,63,64]. In Ghana, three quarters of pharmacies had designated counseling areas compared to 19% of LCSs [65]. Patient history taking was observed to be poorly done in a number of studies. This was seen in Ghana, Nigeria and Sudan [9,56,60]. Nigerian PMVs were more likely to ask questions about illness if the shop was located in a rural setting, if the attending staff was the owner, or if the client was buying the drug for another person [62].

Majority of staff interviewed in Ethiopia, Tanzania, Somaliland, Nigeria and Ghana expressed willingness to refer clients when necessary [26,29,48,61,66]. However, actual referral practices were rarely investigated, and where this was done, the practice was scarcely reported. In Nigeria and Uganda for instance, nearly all staff failed to refer clients when required [11,62]. What made referral particularly hard to assess was poor record keeping, an observation made in Ugandan and Tanzanian drug stores, as well as Ghanaian LCSs [22,65,67]. Pharmacies kept better records , although it was not clear whether this included referral records [65].

So, what factors influence dispensing behavior among SDSs? A number of studies explored determinants of SDS practices across different countries. In Nigeria, 3 separate studies found demand to be an important influence, with 40% of pharmacists and 62-69% of PMVs selling medicines at the request of clients [48,62,68]. Similarly, Ugandan and Tanzanian drug shops sold whichever medicines clients requested [11,43,69], whereas in Kenya and Somaliland, demand was reported to influence pharmacies’ choice of medicines to stock [24,29]. Another important determinant of dispensing practices was shop location, with shops situated in urban and affluent areas providing better services overall. This was seen in Zimbabwe and Kenya for instance. In Zimbabwe, pharmacies located in the capital had a higher likelihood of dispensing correct STI treatments, whereas clients visiting pharmacies located in urban or affluent areas were more likely to receive appropriate treatment in Kenya [58,64]. Additionally, shops located near towns, those operating close to other shops, and shops situated in more populous areas were found to have a higher likelihood of stocking the first line antimalarial treatments in Tanzania, meaning individuals staying within such localities had better geographical access to policy recommended treatments [40]. The same study also found shops operating in remote areas to have a higher likelihood of selling antipyretics in place of antimalarials for fever (the policy at the time was to treat all fevers as malaria cases in high transmission areas). Shop location was associated with medicine prices and credit facilities. In Tanzania, ADDOs operating in more rural areas were found to have higher mark-ups compared to those in urban locations [42], whereas medicines prices were higher in pharmacies operating in more affluent parts of Mozambique’s capital [19]. In Somaliland, four-fifths of rural pharmacies offered credit facilities compared to only half in urban locations [29]. Prices also varied with shop type, with non-pharmacy drug shops having lower prices compared to pharmacies in Nigeria, Uganda, Benin and Zambia [14,31,33,70]. Regulation of retail mark-ups was only encountered in Mali and Mozambique [19,71,72]. Age and sex of client also had some association with dispensing, with women receiving correct diarrhoea treatment more often than men in Kenya, and drug shops expressing higher willingness to sell medicines for sick adults than infants in Tanzania [26,58]. Finally, familiarity with the clients influenced dispensing in Zimbabwe, where one-fifth of pharmacy staff admitted they could sell an antibiotic to an acquaintance without asking for a prescription [64].

### Regulatory and policy issues

Regulatory practices were rarely reported overall. Frequency of regulatory visits varied across countries, with only a third of PMVs in Nigeria reporting visits over the last 2 years in contrast to drug stores in Tanzania, majority of whom had received visits over the last year [28,54]. Low frequency of regulatory inspection was blamed for poor regulatory compliance among pharmacies in Zaire (now the Democratic Republic of Congo), where 87% of drugs were sold without prescription [8]. The sale of unregistered medicines was fairly common in Kenya and Ghana. In Kenya for instance, only 5 out of 12 brands of amodiaquine syrup were registered by the regulatory authority [35], whereas 6-29% of pharmacies and 11-23% of LCSs were found selling various brands of unregistered antimalarials in Ghana [73]. In Tanzania, different patterns were observed across different shop cadres; whereas 19 of 30 drug stores were found stocking unregistered antimalarial imports, nearly all pharmacies were reported to only stock registered malaria medicines [54,74]. Other forms of regulatory infringement included providing clinical management without authorization in Uganda, and failure to display practice permits in Tanzania [54,75]. Drug policy issues were poorly communicated in Tanzania overall, with three-quarters of dispensers reporting that they heard about malaria treatment policy changes over the radio and other news media [7]. This may partly explain the high availability of unrecommended treatments such as artemisinin monotherapies in Tanzanian pharmacies and drug stores [74,76].

## Discussion

The role of SDSs in public health has expanded over recent years, resulting in an overall increase in studies looking at various dimensions of their characteristics and performance, and ways of improving their impact. A recent increase in studies on the topic was demonstrated in the review, with nearly two thirds coming over the last five years. The surge can be explained in part by the fact that the Tanzanian authorities allowed upgrading of part II drug stores into ADDOs, thus resulting in the relatively high number of studies seen from Tanzania.

Majority of studies came from Anglophone countries, possibly due to any one of two reasons; a weakness in the search strategy, or an overall lack of interest in SDSs by the biomedical communities in Francophone countries. Similar concerns have been expressed in previous reviews in SSA [2,6]. Majority of studies were cross-sectional descriptive surveys employing provider questionnaires and/or simulated clients for data collection. Simulated client surveys have gained popularity over recent years because of the ability to minimize bias [77,78]. Studies included in the review were highly heterogeneous, reporting a wide range of outcomes including characteristics of staff and shops, knowledge, and reported and observed practices. This made it impossible to pool estimates or assess overall patterns using any quantitative methods. As a result, a structured narrative synthesis was undertaken to describe the findings from the studies. However, unlike the more rigorous meta-analysis approach, the narrative synthesis approach gives each study equal weight, regardless of sample size and methodological rigour. Consequently, the risk of bias can be expected to be higher in this type of review, thus calling for a higher degree of caution when interpreting findings. In an attempt to minimize this, we placed more emphasis on discussing patterns that emerged across a number of studies and/countries.

Overall, the presence of SDSs correlated positively with population density, with majority operating either in urban areas, or rural areas with larger populations. Incidentally, these also happen to be areas with better health facility networks [79,80]. As far as prices are concerned, no consistent patterns were observed across the countries. In some areas like Tanzania, ADDOs operating in rural areas reportedly charged higher prices for medicines in some areas, presumably to cater for transport costs and compensate for low business activity (although higher competition may also explain lower prices in urban locations). On the other hand, non-pharmacy drug shops (usually found more in rural locations) were reported to charge lower prices compared to the more urban location-linked pharmacies across a number of countries. This observation may reflect a number of factors, including higher purchasing power among the urban clientele, and higher operational costs in the usually larger urban shops. More research needs to explore reasons behind price variations, and find interventions that may reduce such disparities. This is particularly important in the context of the roll-out of Global Fund supported AMFm program, where prices are expected to be standard across all subsidized artemisinin combination therapies (ACTs) [81,82].

Two outcomes with important policy implications are staff turnover and qualifications. Overall, low staff turnover can be expected to maximize benefits of introducing interventions such as training. Evidence points at relatively low staff turnover, particularly among drug shops and PMVs. It is not clear why this is the case, although low capital base has been cited previously as one possible reason [2]. Another possibility stems from the fact that the lower cadre shops are more often manned by owners compared to pharmacies. Of greater concern perhaps, is the issue of unqualified staff serving clients, even where SDSs carry registration certificates from qualified persons. Although reported across all shop cadres, this behaviour was particularly dire among PMVs, some of whom went as far as employing school going children to dispense medicines. Creation of lower practice levels such as part II drug shops and allowing non-pharmaceutical personnel to run SDSs are viewed as policy responses to low numbers of pharmacists; thus meant to ensure better access to medicines and health information. However, the success of the strategy rides on the expectation that license holders (usually people with some health-related training) be available at the premises at all times. The question is; what happens to the quality of care when lower-level providers delegate dispensing responsibilities further? Reliable information on characteristics of staff manning SDSs is vital if policymakers are to make informed decisions on what roles these providers should play. Regrettably though, this knowledge remains inadequate, a view expressed in a previous review on a related subject [2].

Staff knowledge was fairly good on areas such as drug selection, but relatively poor on the more technical aspects such as drug dosages and side-effects, particularly among non-pharmacy drug shops. This is understandable, considering such shops are usually licensed to only sell pre-packaged medicines. However, the role of non-pharmacy SDSs has been gradually expanding over recent years, with Tanzania for instance, relicensing part II drug stores into ADDOs, and granting the accredited shops permission to sell a wider range of medicines. This trend is likely to spread to other areas, particularly following the roll out of the AMFm programme. If the role of SDSs is to be bumped up without compromising quality, comprehensive training approaches are vital. This will ensure misconceptions about medicines are addressed (see for instance, Rutebemberwa *et al* on how auditory pain, a side-effects of quinine, was perceived to be an indicator of how strong the drug was in Uganda, and Dodoo *et al* on how fear of side-effects of amodiaquine made clients prefer artemisinin monotherapy over amodiaquine-containing ACTs in Ghana) [83,84]. The observation that practice did not always reflect knowledge suggests that factors beyond staff awareness are equally important in influencing practices. Unfortunately, interventions for improving the quality of services provided by SDS almost always entail training and related strategies only [5,6]. More studies need to describe other factors, and how interventions can be designed to achieve the highest quality. Goel *et al,* for instance, identified client demand/expectations, physician practice and local regulatory factors as potential determinants of pharmacy staff behaviour [85]. In this review, demand was found to strongly influence drug selection and dispensing. This can be inferred as resulting from market forces, and presents a unique opportunity for policy drivers to influence SDS behaviour through manipulating the demand side preferences. This can be achieved using mass media campaigns targeting perverse community behaviours such as taking incomplete doses of antibiotics. The idea of manipulating preferences to influence private sector behaviour has been reported to be effective in some settings [1].

Shop location was associated with several practices, with clients visiting pharmacies in larger towns or affluent areas receiving better care compared to those in smaller towns or poorer areas. This may have resulted because individuals living in poorer areas had less knowledge on drugs (thus more likely to buy wrong medicines), or it could be an indication of skewed distribution of trained staff in favour of urban and affluent areas. However, such disparities may also occur if staff in urban and affluent areas get more continuous medical training opportunities overall. The observation that rural shops offered credit more readily may reflect better familiarity with the clients, or lower purchasing power overall. This nonetheless points at differences in the nature of relationships between SDSs and clients across the two locations, thus underscoring the value of recognizing rural-urban differences when designing interventions to improve practice.

The high willingness to refer clients reported in some studies is encouraging, although actual referral practices were rarely ascertained in practice. Willingness to refer clients where required presents an opportunity for collaborative partnerships between SDS and health facilities. A similar arrangement formed the basis of a considerably successful programme aimed at improving community management of TB in Bolivia [86]. Such partnerships may also expand the role of SDSs towards preventive services, thus addressing concerns that retailers over provide curative commodities at the expense of advising and counselling clients on broader aspects of health [29].

Few studies looked at regulatory factors overall. Where this was done, the frequency of regulatory visits and severity of sanctions were linked to regulatory compliance, with lower frequency and less severe sanctions being blamed for poor compliance. However, little has been done to examine the exact relationship between regulatory enforcement and SDS performance overall, an observation also made nearly a decade and a half ago [85]. Concerns over evidence gaps on effectiveness of regulation have been expressed in a previous review on SDSs [6]. Finally, proper knowledge is required on the retail sector, particularly considering their growing role in public health. Furthermore, public health interventions appear to be increasingly looking beyond the traditionally legitimated categorization of providers, recognizing instead the blurred boundaries between informal and formal providers, and the fluid nature of line separating legitimate and illegitimate practitioners [87]. As lower levels of practice become increasingly embraced, information on performance and determinants of SDS practices is required to inform policy on allocating appropriate roles and designing interventions to improve their practice.

## Conclusion

Characteristics and practices of SDSs vary across countries and shop cadres. Overall, it appears that the practice of unqualified staff manning shops is common, even where shops carry legitimate licenses. Although the design of pharmacies allowed for better communication with clients, this was rarely done overall. Non-pharmacy drug shop premises were generally more basic in structure. Differences in the structure and location of SDSs should be considered when designing policy interventions aimed at expanding their roles. Similarly, regulations should be responsive to differences in the size, location and nature of business of pharmacies and non-pharmacy drug shops. Whereas majority of staff performed had basic knowledge on drug choice, knowledge on more technical, but equally useful aspects such as side-effects was generally poor, particularly among non-pharmacy shops. As this is likely to be a result of non-qualified staff manning SDSs, more innovative ways of implementing regulatory enforcement need to be developed. With regard to dispensing practices, evidence points at client demand as an important influence, meaning it would be difficult to influence dispensing practices without involving certain aspects of public awareness. Strategies for manipulating demand-side preferences such as social marketing and targeted subsidies could generate additional benefit if included as part of interventions for improving dispensing practices of SDS.

## Competing interests

No competing interests declared.

## Authors' contributions

FW and EM conducted the literature searches and screening. All three authors participated in retrieving articles, conducting the review, and drafting of the manuscript. All authors read and approved the final manuscript.

## Pre-publication history

The pre-publication history for this paper can be accessed here:

http://www.biomedcentral.com/1472-6963/12/223/prepub
